# Comparative evaluation of the stepped wedge design efficiency

**DOI:** 10.1186/1745-6215-14-S1-O46

**Published:** 2013-11-29

**Authors:** Gianluca Baio, Rumana Omar, Gareth Ambler

**Affiliations:** 1 University College London, London, UK

## 

The cluster randomised trial (CRT) design is widely used to evaluate interventions targeted at the cluster level and helps alleviate contamination within clusters. CRTs usually require large sample sizes, although matched pair CRTs can limit this problem. The stepped wedge design (SWD) extends CRTs, in cases where it is expected that the intervention will do more good than harm and/or, for logistical, practical or financial reasons, it is impossible to deliver the intervention simultaneously to all clusters. In a SWD different clusters cross over from control to treatment unidirectionally at different time points. Clusters receive the intervention sequentially over a number of time periods. All clusters are initially assigned to the control condition. At successive, randomly determined times some clusters begin the treatment and the response is measured. Eventually, all receive the treatment.

The literature on SWD is not extensive and thus its relative performance (eg against standard or matched pair CRTs) has not been investigated adequately. The SWD has the potential to reduce the sample size substantially and could be a viable alternative to CRTs provided that the relevant assumptions hold. We perform simulation studies, under different scenarios (eg varying the number of clusters, time periods within the SWD and cluster size, considering both repeated cross-sectional and longitudinal design, for continuous, binary and count data) to identify situations in which the SWD could offer substantial advantages in terms of sample size, without incurring additional costs due to increase in the total length of time to run a trial.

